# Detecting, quantifying and adjusting for publication bias in meta-analyses: protocol of a systematic review on methods

**DOI:** 10.1186/2046-4053-2-60

**Published:** 2013-07-25

**Authors:** Katharina Felicitas Mueller, Joerg J Meerpohl, Matthias Briel, Gerd Antes, Erik von Elm, Britta Lang, Viktoria Gloy, Edith Motschall, Guido Schwarzer, Dirk Bassler

**Affiliations:** 1 Center for Pediatric Clinical Studies, University Children’s Hospital Tuebingen, Frondsbergstraße 23, 72070 Tuebingen, Germany; 2 Department of Neonatology, University Children’s Hospital Tuebingen, Calwer-Str. 7, 72076 Tuebingen, Germany; 3 German Cochrane Centre, Institute of Medical Biometry and Medical Informatics, University Medical Center Freiburg, Berliner Allee 29, 79110 Freiburg, Germany; 4 Basel Institute for Clinical Epidemiology and Biostatistics, University Hospital Basel, Hebelstrasse 10, 3.Etage, 4031 Basel, Switzerland; 5 Cochrane Switzerland, IUMSP, University Hospital Lausanne, Biopôle 2, Route de la Corniche 10, 1010 Lausanne, Switzerland; 6 Institute of Medical Biometry and Medical Informatics, University Medical Center Freiburg, Stefan-Meier-Str. 26, 79104 Freiburg, Germany

**Keywords:** Publication bias, Full publication, Underreporting, Detecting, Quantifying, The OPEN project

## Abstract

**Background:**

Health professionals and policymakers aspire to make healthcare decisions based on the entire relevant research evidence. This, however, can rarely be achieved because a considerable amount of research findings are not published, especially in case of ‘negative’ results - a phenomenon widely recognized as publication bias. Different methods of detecting, quantifying and adjusting for publication bias in meta-analyses have been described in the literature, such as graphical approaches and formal statistical tests to detect publication bias, and statistical approaches to modify effect sizes to adjust a pooled estimate when the presence of publication bias is suspected. An up-to-date systematic review of the existing methods is lacking.

**Methods/design:**

The objectives of this systematic review are as follows:

• To systematically review methodological articles which focus on non-publication of studies and to describe methods of detecting and/or quantifying and/or adjusting for publication bias in meta-analyses.

• To appraise strengths and weaknesses of methods, the resources they require, and the conditions under which the method could be used, based on findings of included studies.

We will systematically search Web of Science, Medline, and the Cochrane Library for methodological articles that describe at least one method of detecting and/or quantifying and/or adjusting for publication bias in meta-analyses. A dedicated data extraction form is developed and pilot-tested. Working in teams of two, we will independently extract relevant information from each eligible article. As this will be a qualitative systematic review, data reporting will involve a descriptive summary.

**Discussion:**

Results are expected to be publicly available in mid 2013. This systematic review together with the results of other systematic reviews of the OPEN project (To Overcome Failure to Publish Negative Findings) will serve as a basis for the development of future policies and guidelines regarding the assessment and handling of publication bias in meta-analyses.

## Background

Syntheses of published research, such as meta-analyses and systematic reviews, are becoming increasingly important in providing relevant and valid research evidence to clinical and health policy decision making. However, published studies might represent a biased selection of all studies that have been conducted, if statistically significant or ‘positive’ results are published preferably, a phenomenon widely known as publication bias [1-4]. When searching the literature for meta-analyses, unpublished studies and studies published in the so called ‘grey literature’ only (such as conference abstracts, dissertations, policy documents, and book chapters) might be missed. The effect estimates of meta-analyses based exclusively on the published literature might be exaggerated and represent an overestimation of the true effect size [2,5], and consequently the patient might be exposed to an ineffective or even harmful treatment.

Unfortunately, the elimination of publication bias can seldom be achieved, since relevant ‘unpublished’ studies are frequently difficult to find or not accessible. There are basically two kinds of ‘unpublished’ data. The first type of data, described as grey literature in the paragraph above, can still be identified through extended search strategies in computerized databases. The second type refers to data that have not been published at all and thus are far more difficult to identify. In order to tackle bias related to non-publication or distortion in the publication process of study findings there have been various calls for mandatory registration of clinical trials at inception [6]. In 2004, major medical journals agreed that they would only publish trials that were previously registered [7]. However, some of the data fields requested in the registries are frequently incomplete [8]. Thus, until the complete registration at inception of all trials is a well-established method and results of all trials are publicly available, it is of great importance to improve methods for detection, quantification and the adjustment for publication bias in meta-analyses and systematic reviews.

In the literature various methods to detect, quantify and adjust for publication bias in meta-analyses have been described. There are graphical approaches, such as funnel plots [9], formal statistical tests to detect the presence of publication bias, such as the regression test proposed by Egger and colleagues [9], and statistical approaches to modify effect size to adjust pooled estimates when the presence of publication bias is suspected, such as the trim-and-fill method [10]. Still, statistical approaches to correct for missing studies are precarious. For instance, some authors criticize that the visual interpretation of a funnel plot depends too much on the subjective impression of the observer [11,12]. Furthermore, the performances of many of these methods have been evaluated using simulation studies, but concerns remain as to whether the simulations reflect real-life situations.

Currently, consensus on what method is best to use only exists for the special case of tests for funnel plot asymmetry in meta-analyses of randomized controlled trials [13]. In order to inform the future development of policies and guidelines regarding the assessment of publication bias, we will conduct a systematic review of methods described in the literature.

### Objectives

•To systematically review methodological articles which focus on non-publication of studies and to describe methods of detecting and/or quantifying and/or adjusting for publication bias in meta-analyses.

•To appraise strengths and weaknesses of methods, the resources they require, and the conditions under which the method could be used, based on findings of included studies.

This systematic review will be part of the OPEN project (To Overcome Failure to Publish Negative Findings) which, among other objectives, aims to elucidate the non-publication of studies through a series of systematic reviews [14].

## Methods

### Search strategy

Literature searches for methodological studies are often difficult because of ill-defined boundaries and inappropriate indexing in commonly used bibliographic databases [15]. Previous experience by Song and colleagues [16] suggests that the most productive and efficient methods include searching the Cochrane Methodology Register. In addition to the suggested Cochrane Methodology Register, we will conduct electronic literature search in Web of Science and Medline to identify relevant methodological articles. Methodological articles are those that have developed or investigated methods for detecting, quantifying or adjusting for publication bias. As the MeSH term “publication bias” has been introduced only in 1994, we decided to restrict our literature search to articles published between 1994 and the present to facilitate our literature search. Key words used in the search of electronic databases will include: publication bias, file-drawer, and reporting bias. The search strategy has been developed with the support of a librarian/information specialist and we will focus on fully published articles. We will ask experts in the field for any additional references and check references of included articles. No language restrictions will be applied. The full search strategies are displayed in Additional file 1.

### Eligibility criteria

Methodological articles will be considered eligible for inclusion if they describe a method for at least one of the following tasks: i) detection, ii) quantification, or iii) adjustment for publication bias in meta-analyses.

We will exclude original clinical trial reports, observational clinical studies, and clinical systematic reviews.

### Study selection

Two reviewers will independently and in duplicate screen titles and abstracts of search results. If both reviewers do not agree on exclusion based on its title and abstract, the full text will be retrieved and assessed for eligibility. Any disagreement among reviewers will be resolved by discussion and consensus or, if needed, arbitration by a third reviewer.

### Data extraction

A dedicated data extraction form will be used (Additional file 2), and two reviewers will independently extract the following information:

○ Basic data:

○ author names

○ language

○ year of publication

○ journal name

○ type of report (for example, narrative review,

systematic review, methodological study and so on)

○ study objectives

○ funding source

○ On methods to detect and/or quantify and/or

adjust for publication bias in meta-analyses:

○ Short description of the method

○ What form of bias the method pays attention to

○ Underlying assumptions

○ Purpose of the method

○ Resources required for using the method

○ Strengths and weaknesses of the methods

described (as discussed in the article)

○ If the method has been applied to meta-analyses with real world datasets, or to a dataset for which one can be reasonably confident that all studies conducted have been included (for example, datasets from trial registries from medical regulatory authorities such as the Food and Drug Administration (FDA) yes the definition is correct or the European Medicines Agency (EMA)).

The full data extraction sheet is displayed in Additional file 2.

Two reviewers will extract relevant data from each of the included methodological articles independently and in duplicate. Any disagreements will be resolved by discussion and consensus or, if needed, arbitration by a third reviewer.

### Data analysis and reporting

A short definition of the method described to detect and/or quantify and/or adjust for publication bias in meta-analyses will be given. Each method will be classified as described by the author. If the author does not propose a classification, we will categorize the method based on a standardized method classification sheet.

The different categories comprise:

•Study registration

•Literature search

•Funnel plot

○ Tests for funnel plot asymmetry

○ Methods to adjust for publication bias based on funnel plots (trim and fill)

•Selection models

•Selection models with data augmentation

•Sensitivity analyses based on selection models

•New statistical approaches

•Updating reviews

•Publication process

•Research ethics/policy

•Confirmatory studies

•Other: _____________

Available methods will be critically appraised in terms of underlying assumptions, conditions under which the method could be used, usefulness, limitations, and resources required. This appraisal will be based on the description provided in the included studies.

To assess the validity of the method, we will describe if the method has been tested in an empirical dataset for which one can be reasonably confident that all studies conducted have been included (for example, datasets from trial registries from medical regulatory authorities such as the FDA or EMA).

Data extracted from the included studies and results of critical appraisal will be presented in tables and described narratively. We will report this systematic review according to PRISMA (Preferred Reporting Items for Systematic Reviews and Meta-Analyses) guidelines [17].

## Discussion

We aim to provide a comprehensive systematic review of the various methods to detect and/or quantify and/or adjust for publication bias, as they are used and described in the literature. Furthermore, we will describe the various methods found and illustrate the strengths and weaknesses of each method in this systematic review.

Our protocol has strengths and limitations. A strength of our protocol is the systematic approach to identify methodological articles through a sensitive search strategy, which includes databases previously tested for the search of methodological studies on publication bias [16]. A limitation of this protocol is the assessment of the various methods based only on the information provided in the methodological articles themselves without applying them to a real world clinical dataset.

As part of the OPEN project, this systematic review aims to raise awareness of the importance of bias related to non-publication or distortion in the publication process of research findings and the complexity of this issue. This, and other systematic reviews conducted in the OPEN project, will also provide a foundation for a recommendations workshop, during which key members of the biomedical research community (for example, funders, research ethics committees, journal editors) will develop future policies and guidelines to tackle the non-publication of biomedical research findings and related biases.

## Abbreviations

EMA: European Medicines Agency; FDA: Food and Drug Administration; OPEN: to overcome failure to publish negative findings; PRISMA: Preferred reporting items for systematic reviews and meta-analyses.

## Competing interests

We declare that all authors and contributing members have no competing interests.

## Authors’ contributions

DB and JJM conceived the study. MB and EM designed the search strategies. KFM drafted the manuscript with the help of DB. KFM, JJM, MB, GA, EvE, BL, VG, EM, GS and DB critically reviewed the manuscript for important intellectual content. KFM and DB are guarantors. All authors read and approved the final manuscript.

## Supplementary Material

Additional file 1 Search results and search strategies. Click here for file

Additional file 2 Data extraction sheet. Click here for file

## References

[B1] Dickersin K Min YI NIH clinical trials and publication bias Online J Curr Clin Trials 1993 Doc No 50: [4967 words; 53 paragraphs]. http://www.ncbi.nlm.nih.gov/pubmed/8306005 8306005

[B2] Easterbrook PJ Berlin JA Gopalan R Matthews DR Publication bias in clinical research Lancet 1991 337 867 872 10.1016/0140-6736(91)90201-Y 1672966

[B3] Dickersin K The existence of publication bias and risk factors for its occurrence JAMA 1990 263 1385 1389 10.1001/jama.1990.03440100097014 2406472

[B4] Dwan K Altman DG Arnaiz JA Bloom J Chan AW Cronin E Decullier E Easterbrook PJ Von Elm E Gamble C Ghersi D Ioannidis JP Simes J Williamson PR Systematic review of the empirical evidence of study publication bias and outcome reporting bias PLoS One 2008 3 e3081 10.1371/journal.pone.0003081 18769481 PMC2518111

[B5] Song F Eastwood AJ Gilbody S Duley L Sutton AJ Publication and related biases Health Technol Assess 2000 4 1 115 10932019

[B6] Simes RJ Publication bias: the case for an international registry of clinical trials J Clin Oncol 1986 4 1529 1541 3760920 10.1200/JCO.1986.4.10.1529

[B7] Abbasi K Compulsory registration of clinical trials BMJ 2004 329 637 638 10.1136/bmj.329.7467.637 15374895 PMC517630

[B8] Zarin DA Tse T Ide NC Trial Registration at ClinicalTrials.gov between May and October 2005 N Engl J Med 2005 353 2779 2787 10.1056/NEJMsa053234 16382064 PMC1568386

[B9] Egger M Davey Smith G Schneider M Minder C Bias in meta-analysis detected by a simple, graphical test BMJ 1997 315 629 634 10.1136/bmj.315.7109.629 9310563 PMC2127453

[B10] Duval S Tweedie R Trim and fill: A simple funnel-plot-based method of testing and adjusting for publication bias in meta-analysis Biometrics 2000 56 455 463 10.1111/j.0006-341X.2000.00455.x 10877304

[B11] Greenland S Invited commentary: a critical look at some popular meta-analytic methods Am J Epidemiol 1994 140 290 296 8030632 10.1093/oxfordjournals.aje.a117248

[B12] Terrin N Schmid CH Lau J In an empirical evaluation of the funnel plot, researchers could not visually identify publication bias J Clin Epidemiol 2005 58 894 901 10.1016/j.jclinepi.2005.01.006 16085192

[B13] Sterne JA Sutton AJ Ioannidis JP Terrin N Jones DR Lau J Carpenter J Rucker G Harbord RM Schmid CH Tetzlaff J Deeks JJ Peters J Macaskill P Schwarzer G Duval S Altman DG Moher D Higgins JP Recommendations for examining and interpreting funnel plot asymmetry in meta-analyses of randomised controlled trials BMJ 2011 343 d4002 10.1136/bmj.d4002 21784880

[B14] Portalupi S Von Elm E Schmucker C Lang B Motschall E Schwarzer G Gross IT Scherer RW Bassler D Meerpohl JJ Protocol for a systematic review on the extent of non-publication of research studies and associated study characteristics Systematic Reviews 2013 2 2 10.1186/2046-4053-2-2 23302739 PMC3577647

[B15] Edwards SJL Lilford RJ Kiauka S Black N, Brazier J, Fitzpatrick R, Reeves B Different types of systematic review in health services research Health Services Research Methods: a Guide to Best Practice 1998 London: BMJ Books 255 259

[B16] Song F Parekh S Hooper L Loke YK Ryder J Sutton AJ Hing C Kwok CS Pang C Harvey I Dissemination and publication of research findings: an updated review of related biases Health Technol Assess 2010 14 1 193 20181324 10.3310/hta14080

[B17] Moher D Liberati A Tetzlaff J Altman DG Group P Preferred reporting items for systematic reviews and meta-analyses: the PRISMA statement Ann Intern Med 2009 151 264 269 10.7326/0003-4819-151-4-200908180-00135 19622511

