# Supplementary material for: Detecting, quantifying and adjusting for publication bias in meta-analyses: protocol of a systematic review on methods
Source: Syst Rev. 2013 Jul 25;2:60. doi: 10.1186/2046-4053-2-60 (PMC3733739; doi:10.1186/2046-4053-2-60)
Supplement: Additional file 1 — Search results and search strategies. [file 2046-4053-2-60-S1.doc]

***Additional file 1*: Search results and search strategies**

Databases Publication Bias Methods

| **Database** | **Provider** | **Time Span** |
| --- | --- | --- |
| Web of Science:  Science Citation Index,  Social Science Citation Index | Thomson Reuters | 1994 - current |
| Cochrane Library:  Methodology Reviews | Wiley | 1994 - current |
| Cochrane Library:  Methods Studies |  | 1994 - current |
| Medline | OvidSP | 1994-current |
| Medline Daily Update |  | Search day |

**Web of Science Search Strategy (1994-current)**

Timespan = 1994-01-01 – present

Databases = Science Citation Index, Social Science Citation Index

Lemmatization = Off (Alternative forms of search terms are not automatically applied)

| No. | Query |
| --- | --- |
| #1 | ti=("publication bias*" or "file draw* bias*" or "report* bias*" or "meta-bias*" or metabias*) |

Ti = title

* = Truncation

**Cochrane Library Search Strategy (1994-current )**

Methodology Reviews: Issue 12 (Dec) 2012

Method Studies: Issue 4 (Oct) 2012

| ID | Search |
| --- | --- |
| #1 | ((publication near bias*) or "reporting bias*"):ti,ab,kw from 1994 to 2013, in Methods Studies |
| #2 | ((publication near bias*) or "reporting bias*"):ti,ab,kw from 1994 to 2013, in Cochrane Reviews (Reviews only) |

Ti= title, ab = abstract, kw=keywords

* = truncation

Near = within 6 words in any order

**Ovid Medline Search Strategy (1994- present)**

| # | Searches |
| --- | --- |
| 1 | *publication bias/ |
| 2 | (publication bias* or file draw* bias* or report* bias* or meta-bias* or metabias*).ti. |
| 3 | 1 or 2 |
| 4 | limit 3 to yr="1994 -Current" |
| 5 | remove duplicates from 4 |

/ = Medical Subject Heading

**Medical Subject Heading/*  = Focus (Major Topic)

* = Truncation

ti. = title

yr = publication year
