# Supplementary material for: Detecting, quantifying and adjusting for publication bias in meta-analyses: protocol of a systematic review on methods
Source: Syst Rev. 2013 Jul 25;2:60. doi: 10.1186/2046-4053-2-60 (PMC3733739; doi:10.1186/2046-4053-2-60)
Supplement: Additional file 2 — Data extraction sheet. [file 2046-4053-2-60-S2.doc]

***Additional file 2*: Data extraction sheet**

**I. Full text eligibility assessment**

Inclusion criteria:

O Methodological articles that describe a method on at least one of the following:

i) detection, ii) quantification, iii) adjustment for publication bias in meta-analyses.

Exclusion criteria:

O Original trial reports

O Observational studies

O Systematic reviews with a clinical focus

If they have detected and/or quantified and/or adjusted for publication bias only in their very specific field.

Decision:

O Included

O Excluded, because: ________________________________________________

**II. Data extraction sheet for: Detecting, quantifying and adjusting for publication bias in meta-analyses: a methods overview**

**Baseline Data:**

Article Number:

Author (year):

Language:

Title:

Journal:

Type of report:

O Abstract

O Commentary / Editorial

O Guidelines / HTA – report

O Methodological study

O Narrative Review

O Systematic Review

O Other: __________________________

Funding Source:

O Governmental / public

O Charity / private not for profit

O Industry / private for profit

O Not funded / only in house source

O Not reported

O Reported, but type of funding unclear

**Study objectives:**

O New method

O Established method

O Evidence of usefulness/limitations

O Extent of publication bias

O Comparison of various methods

**Underlying assumptions:**

O True treatment effect is zero

O True treatment effect is small or moderate but not zero

O True treatment effect is big

O Not described

O Other: ________________________________________________

**Method:**

**Name of the method, as proposed by the author:** _______________________________

**Short description of the method:**

**Purpose of the method:**

O Detecting bias

O Quantifying bias

O Adjusting / Reducing bias

O Preventing bias

O Other: _______________________

**Classification of method, as proposed by the author:**

O Study registration

O Literature search

O Funnel plot

O Tests for tunnel plot asymmetry

O Methods to adjust for publication bias based on funnel plots (trim and fill)

O Selection models

O Selection models with data augmentation

O Sensitivity analyses based on selection models

O New statistical approaches

O Updating reviews

O Publication process

O Research ethics/policy

O Confirmatory studies

O Other: _____________

**If no assignment by author made, method could be classified as:**

O Study registration

O Literature search

O Funnel plot

O Tests for tunnel plot asymmetry

O Methods to adjust for publication bias based on funnel plots (trim and fill)

O Selection models

O Selection models with data augmentation

O Sensitivity analyses based on selection models

O New statistical approaches

O Updating reviews

O Publication process

O Research ethics/policy

O Confirmatory studies

O Classification not possible

O Other: _____________

**On what stage of the literature review process the method is relevant:**

O Before literature review

O In literature review

O After literature review

**What form of bias the method pays attention to:**

O Publication bias

O Grey literature bias

O Language bias

O Reporting bias

O Abstract bias

O Time delay bias

O Database index bias

O Citation bias

O Duplicate bias

O Media attention bias

O Reporting bias

O Other: ________________

**Any definition given for publication bias:**

**Resources required to use the method:**

O Special software

O Costs, how much:

O Statistical expert

O Time, how much:

O Other: _______________________________

O not described

**Pros and Cons of the method, as described by the author:**

O not described by the author

**Pros:**

**Cons:**

**Compared to what other method:**

O Study registration

O Literature search

O Funnel plot

O Tests for tunnel plot asymmetry

O Methods to adjust for publication bias based on funnel plots (trim and fill)

O Selection models

O Selection models with data augmentation

O Sensitivity analyses based on selection models

O New statistical approaches

O Updating reviews

O Publication process

O Research ethics/policy

O Confirmatory studies

O Other: _____________

O None

**Best group to use the method for, as described by the author:**

O Not described

O Large number of studies (>100)

O No significant heterogeneity in the studies included

O Only for continuous outcomes

O Only for dichotomous outcomes

O Other: __________________________________

**Method has been applied in a meta-analysis / systematic review of real world data:**

O Not reported

O Yes, with success

O Yes, without success

O Yes, with no report of the result

O If Yes in which data – set

O clinical

O not – clinical

O Yes, in an empirical dataset for which one can be reasonably confident that all studies conducted have been included (e.g. datasets from trial registries from medical regulatory authorities such as FDA or EMA).

O No

**Reviewer’s commentary** (e.g. on study’s validity, scientific rigour, method’s usefulness and limitations, any empirical evidence provided):
